# The Common Task

**Published:** 1908-07-25

**Authors:** 


### THE COMMON TASK.

### The Masseuses' Pocket-book.

We can cordially recommend this little volume by Araminta Ross (Scientific Press; price 2s. net, 2s. 2d. post free) as containing in portable form that concise information about bones, nerves, and muscles which it is essential for the masseuse to have always in mind.

### Visiting Nurses.

The career of a visiting nurse has many attractions for those who like variety, and dread, above all things, the curbing hand of a committee. It appears to offer the maximum of liberty and all the interests of private nursing with the comfort of home life. Yet it has serious drawbacks, and not the least of these is the extent to which the visiting nurse drifts—in the suburbs, at any rate—into taking emergency night work. If her daily engagements are sufficiently numerous she may, of course, decline this branch of work; but when, as often happens, she fails to get her time filled up, she is driven back upon this unsatisfactory resource, and unless she risks losing her connection is exposed to the danger of never having an undisturbed moment night or day. It is infinitely better that night nurses should be supplied from an institute, where precautions can be taken against the worry of mingling day and night work in the person of one nurse. From the point of view of earning an income, the visiting nurse's career is one of the most arduous and precarious that the profession offers. It should never be chosen single-handed or without influential support.

### Medical Inspection in Schools.

It is earnestly to be hoped that any sums which the municipal authorities may dedicate to the nursing service, which is an indispensable part of the new medical inspection of schools, will be secured for the district and village nurses, and not be ear-marked for creating a new order of visiting school inspectors. The village nurse knows all the children in the place, and has probably brought many of them into the world. She has their confidence and that of their mothers, and in the event of special treatment being required as a consequence of the medical inspection she is the proper person to see that it is carried out. Her place could ill be filled by a county nurse, incessantly occupied in travelling about and seeing the children but once in a year or two. There is great danger that the medical inspection in country districts will fail in its effects for want of anyone whose business it is to follow it up. This danger can only be avoided by the presence of the district nurse, who, when once she is aware of defects in the children, can bring the necessity for precaution and simple treatment constantly before the parents. The heavy strain which the maintenance of a nurse entails in some districts would be considerably lightened if the authorities contributed in fair proportion to the funds in return for public services rendered by the nurse.

THE HOSPITAL July 25, 1908.

